# Maternal Micronutrient Supplementation and Long Term Health Impact in Children in Rural Bangladesh

**DOI:** 10.1371/journal.pone.0161294

**Published:** 2016-08-18

**Authors:** Tania Mannan, Sultan Ahmed, Evana Akhtar, Anjan Kumar Roy, Md Ahsanul Haq, Adity Roy, Maria Kippler, Eva-Charlotte Ekström, Yukiko Wagatsuma, Rubhana Raqib

**Affiliations:** 1 Immunobiology, Nutrition and Toxicology Laboratory, Infectious Diseases Division, icddr,b, Dhaka, Bangladesh; 2 Department of Immunology, Bangladesh University of Health Sciences, Mirpur, Dhaka, Bangladesh; 3 Institute of Environmental Medicine, Karolinska Institutet, Stockholm, Sweden; 4 International Maternal and Child Health (IMCH), Department of Women’s and Children’s Health, Uppsala University, Uppsala, Sweden; 5 Department of Clinical Epidemiology, Faculty of Medicine, University of Tsukuba, Tsukuba, Ibaraki, Japan; TNO, NETHERLANDS

## Abstract

**Background:**

Limited data is available on the role of prenatal nutritional status on the health of school-age children. We aimed to determine the impact of maternal micronutrient supplementation on the health status of Bangladeshi children.

**Methods:**

Children (8.6–9.6 years; n = 540) were enrolled from a longitudinal mother-child cohort, where mothers were supplemented daily with either 30mg iron and 400μg folic acid (Fe30F), or 60mg iron and 400μg folic acid (Fe60F), or Fe30F including 15 micronutrients (MM), in rural Matlab. Blood was collected from children to determine the concentration of hemoglobin (Hb) and several micronutrients. Anthropometric and Hb data from these children were also available at 4.5 years of age and mothers at gestational week (GW) 14 and 30.

**Results:**

MM supplementation significantly improved (p≤0.05) body mass index-for-age z-score (BAZ), but not Hb levels, in 9 years old children compared to the Fe30F group. MM supplementation also reduced markers of inflammation (p≤0.05). About 28%, 35% and 23% of the women were found to be anemic at GW14, GW30 and both time points, respectively. The prevalence of anemia was 5% and 15% in 4.5 and 9 years old children, respectively. The adjusted odds of having anemia in 9 year old children was 3-fold higher if their mothers were anemic at both GW14 and GW30 [Odds Ratio (OR) = 3.05; 95% Confidence Interval (CI) 1.42, 6.14, P = 0.002] or even higher if they were also anemic at 4.5 years of age [OR = 5.92; 95% CI 2.64, 13.25; P<0.001].

**Conclusion:**

Maternal micronutrient supplementation imparted beneficial effects on child health. Anemia during pregnancy and early childhood are important risk factors for the occurrence of anemia in school-age children.

## Introduction

A growing body of evidence indicates that the nutritional background of a woman during pregnancy and especially during early life is a critical determinant of her offspring’s subsequent health outcomes such as sub-optimum growth and mortality, suggesting an intergenerational transfer of poor health from mother to child [1,2]. The underlying cause of about 60% of childhood death below 5 years of age in Bangladesh is malnutrition which involves both calorie and micronutrient deficiencies [3]. In this age-group children suffer from one or more forms of malnutrition including stunting (32%), underweight (30%) and anemia (33%) [4]. Iron deficiency and iron deficiency anemia is a major public health problem worldwide, mostly in pregnant women, infants, and young children in developing countries [5]. It adversely affects some vital aspects of human health, including poor cognitive development, decreased immune function and work productivity [5–7]. The most common causes of anemia are nutritional (vitamin and mineral deficiencies such as iron, folate and B vitamins, malnutrition in general) and non-nutritional factors (acute and chronic infections, genetic disorders, poverty, sociocultural and maternal factors) [8]. The majority of the studies reporting maternal anemia as a risk factor for childhood anemia have focused on under-5 children [9,10]. However, the long-term effect of maternal anemia during pregnancy in older school-age children remains unclear.

A large number of the studies emphasized the effects of maternal micronutrient supplementation on pregnancy outcomes, birth outcomes, neonatal/child survival, child growth and morbidity outcomes [11,12]. Data is scarce on the consequence of long-term effects of maternal nutrition supplementation on nutritional status in school going children. In this study, we aimed to evaluate whether maternal micronutrient supplementation during pregnancy influences nutritional and micronutrient status in school-age children (~9 years) in a longitudinal mother-child cohort in rural Bangladesh [13,14]. A secondary aim was to investigate whether anemia during pregnancy has a long-term impact on anemia in pre-adolescent school-age children and whether this is influenced by maternal micronutrient supplementation.

## Methods

### Study area and population

This study is a follow-up of the MINIMat (the Maternal and Infant Nutrition Interventions, Matlab) (ISRCTN16581394) mother-child cohort [13]. It was conducted between 2012 and 2013 in Matlab, a rural area located 53 km southeast of Dhaka, Bangladesh. The icddr,b, runs a health and demographic surveillance system in Matlab, as well as a central hospital and four connected sub-centers that provide health care to the resident population (about 220,000) in the area.

The MINIMat trial included 4436 women identified as pregnant between November 2001 and October 2003 [14]. The enrolled women were randomly assigned to one of three different micronutrient supplementations: (i) 30 mg iron and 400 μg folic acid (Fe30F), (ii) 60 mg iron and 400 μg folic acid (Fe60F), or (iii) the UNICEF preparation of 15 different micronutrients (MM) including 30 mg iron and 400 μg folic acid [14]. Baseline data for mothers were taken by trained health research workers at the first sub-center visit in pregnancy week 8. Hemoglobin (Hb) levels of mothers were measured by HemoCue photometer (HemoCue® AB, Ängelholm, Sweden) during GW14 and GW30. Socioeconomic status (SES) of the families was estimated via an asset score, generated through principal component analysis of household assets and grouped into tertiles [15].

**Fig 1:**
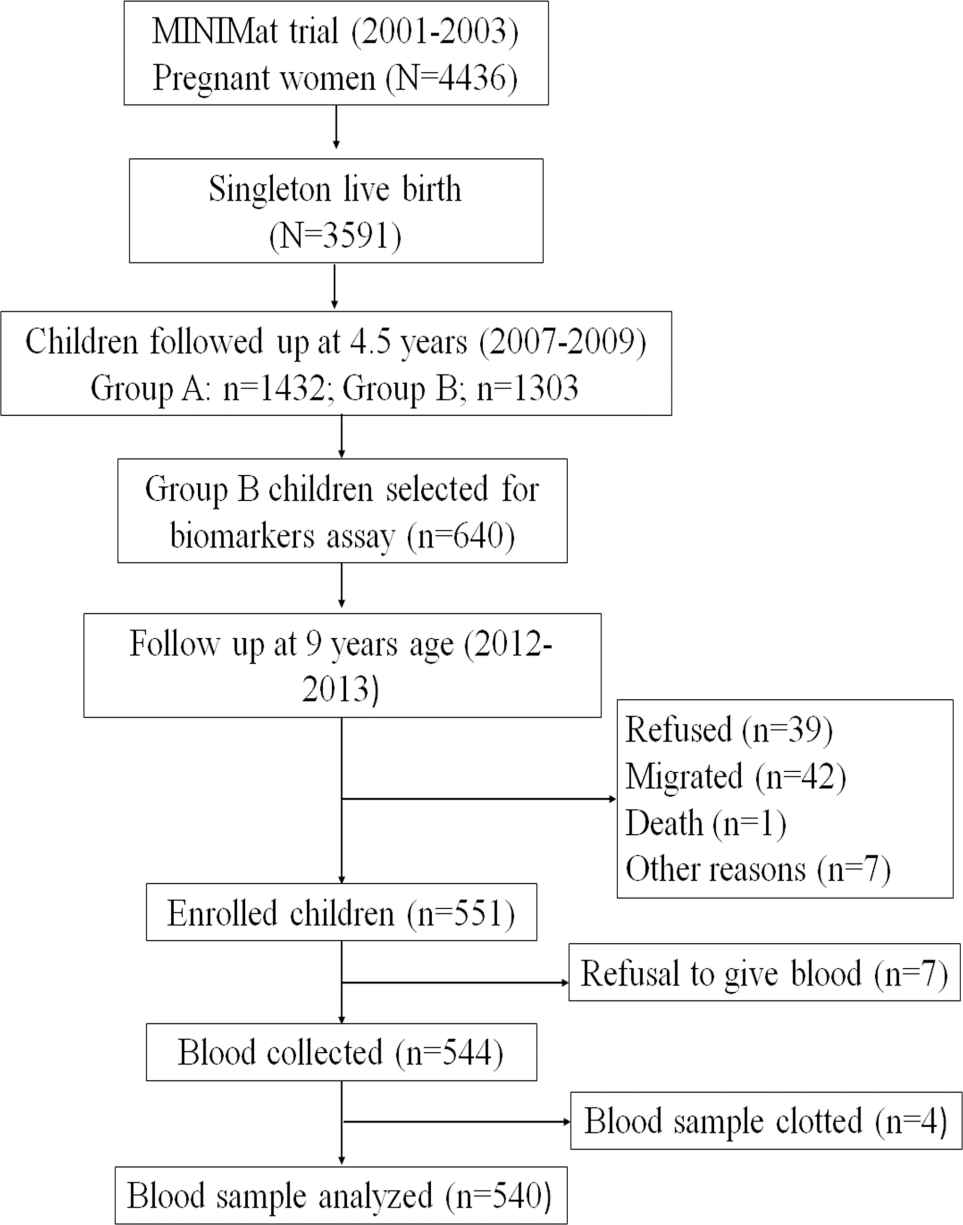
Flow chart describing the enrolment of the children in the current study. The group A children (n = 1432) were born between April 2002 to May 2003 and group B (n = 1303) were born between June 2003 to June 2004.

A total of n = 2735 MINIMat children were studied at the age of 4.5 years. To reduce the burden of various types of investigations, the children were divided into two groups on the basis of the calendar year of birth. Group A children (n = 1432) were born between April 2002 to May 2003. Group B children (n = 1303) were born between June 2003 to June 2004 and were studied for asthma, allergy (total group B children, n = 1303, referred in Hawlader et al) [16,17] as well as immune function and bone growth studies (n = 640 group B children reported in Ahmed et al) [13,18]. These group B children were earlier involved in various studies from birth and at the age of 4.5 years [13,18–22]. Anthropometric data and family SES in 4.5 years old children were determined as described earlier [13]. Hb levels at 4.5 years of age were measured by HemoCue photometer. In the current study, field workers carried out a survey by visiting the household of children studied at 4.5 years of age (n = 640) for availability at 9 years of age [13]. Eighty nine children were not available for various reasons (Fig 1). A total of 551 children were enrolled in the current study, among them 7 children refused to give blood and 4 blood samples were clotted. Finally, 540 blood samples were available for analysis (Fig 1).

Data collection for children at 9 years of age was carried out in a similar way as done for earlier follow up at 4.5 years of age [13]. In brief, anthropometric measurements were taken at the sub-center clinic visit by trained nurses. Weight was measured with a digital scale (TANITA HD-318, Tanita Corporation, Japan), accurate to ±10g. Weight scales were standardized daily. Height was measured using a free standing stadiometer Leicester Height Measure with millimeter marks (Seca214, UK). The stadiometer was calibrated before the start of the study and in every 6 months. The measured weight and height were converted to weight-for-age (WAZ), height-for-age (HAZ) and body mass index (BMI)-for-age Z-scores (BAZ) (standard deviation (SD) scores), using the WHO Multicentre Growth Reference Study child growth standards [23,24]. Children with WAZ <-2 SD from WHO reference population were considered as underweight, with HAZ <-2SD as stunted, and those with BAZ <-2SD were considered as thin. Family SES data were updated as described above.

### Assessment of plasma biomarkers

Five ml of fasting venous blood was collected in Lithium heparin treated tubes (Sarstedt Monevette®, Sweden) by trained paramedics in the subcenters in Matlab. A drop of blood was used to measure Hb by HemoCueHb 201+ (HemoCue AB, Ängelholm, Sweden). The blood samples were transported to the Matlab Laboratory for separation of plasma which was thereafter stored in -80°C freezer until transported to the Laboratory in icddr,b, Dhaka. Ferritin, vitamin B_12_ and folate were analyzed in plasma by chemiluminescence method using Cobas e601 (Roche Diagnostics, Mannheim, Germany). C-reactive protein and sTfR were assessed in plasma using Hitachi 902 (Roche Diagnostics, Mannheim, Germany). Plasma hepcidin was measured using a commercial ELISA kit (DRG International, GmbH, Germany). Flame atomic absorption spectroscopy was used to analyze plasma zinc (Zn) concentrations (Shimadzu Corporation, Kyoto, Japan). Isocratic reverse-phase HPLC and UV detection were used to analyze concentrations of plasma retinol/vitamin A (Shimadzu Corporation, Kyoto, Japan). The mean inter-day coefficient of variation was <5% for sTfR, ferritin, folate, vitamin B_12,_ Zn, vitamin A, and CRP and <8% for hepcidin. For an independent assessment of the laboratory’s analytical performance, it participates in external quality assurance programs such as VITAL EQA of Centers for Disease Control and Prevention (CDC) for the above parameters excepting Zn. The values are given in S1 Table.

The complete blood count (CBC) including mean corpuscular volume (MCV), mean corpuscular Hb (MCH), mean corpuscular Hb concentration (MCHC) and other blood indices were determined using Hematology Analyzer (Sysmex XT-1800i, Kobe, Japan). Erythrocyte Sedimentation Rate (ESR) was measured by Wintrobe Method [25].

### Hb and anemia

Anemia is defined as Hb<115g/l for 9years and <110g/l for <4.5years old children according to WHO guidelines [26] (S2 Table). Mild anemia is defined if Hb level is 110-114g/l, moderate is 80–109 g/l, and severe anemia is <80g/l [27]. The cut-off to define anemia in pregnancy was set to Hb<110 g/l according to WHO [5]. The cut-off values to define iron deficiency (ID), iron deficiency anemia (IDA) [26,28], and micronutrient deficiencies [29–31] have been described in S2 Table.

### Ethics

The institutional review board at icddr,b consists of two review committees, namely the Research Review Committee (RRC) and Ethical Review Committee (ERC). The study was approved by both committees. Written informed consent was obtained from the parents or guardian of each child prior to participation in the study.

### Statistical analyses

Data analyses were done using the Statistical package for the Social Science (SPSS) for Windows (version 20; Armonk, NY: IBM SPSS corp.; 2011) and Stata/IC, version 13 (StataCorp, Texas, USA). Normality (data distribution patterns) and homogeneity of variances were formally checked. Independent sample t-test, Mann-Whitney U-test, ANOVA or Kruskal-Wallis test was used as appropriate for an initial assessment. Analysis of covariance with least significant difference (LSD) was used for multiple comparisons of child nutritional status and plasma biomarkers in relation to supplementation groups. Linear regression analyses were used to evaluate the influence of plasma biomarkers on Hb levels. Multivariable adjusted logistic regression analyses were used to evaluate the risk of having anemia in 9 years old children if they were anemic at 4.5 years of age or their mothers were anemic during pregnancy. Statistical models were adjusted for covariates that were associated with exposure and outcome, or biologically relevant or changed the effect estimate more than 5%. These were SES, mother’s education, mother’s occupation, child BMI, child sex, and plasma CRP. P values <0.05 were considered statistically significant.

## Results

### Anthropometric measurements

Basic characteristics of the children and their mothers were presented in Table 1. At 9 years of age, about 22% children were stunted and 40% were underweight (Table 1). Among the stunted children, 10% were severely stunted (HAZ<-3SD) while among the underweight children, 26% were severely underweight (WAZ<-3SD). About 26% children were thin (Table 1); among them 21% were severely thin (BAZ<-3SD).

**Table 1:** Basic characteristics of mothers and their children.

	All children	Fe30F	Fe60F	MM
	(n = 540)	(n = 188)	(n = 185)	(n = 167)
**Mothers**				
^a^Hb at GW14 (g/l)	116.98±12.70	117.08±11.87	116.69±13.68	117.21±12.50
^b^Hb at GW30 (g/l)	113.98±11.55	113.24±12.45	114.76±11.48	113.92±10.55
**Updated family SES (at 9 years)**				
1^st^ tertile, n (%)	180 (33.46%)	61 (32.45%)	64 (34.59%)	55 (33.33%)
2^nd^ tertile, n (%)	179 (33.27%)	65 (34.57%)	54 (29.19%)	60 (36.36%)
3^rd^ tertile, n (%)	179 (33.27%)	62 (32.98%)	67 (36.22%)	50 (30.30%)
^c^**Education**				
Illiterate, n (%)	59 (11.01%)	13 (6.99%)	24 (12.97%)	22 (13.33%)
Primary level, n (%)	190 (35.45%)	72 (38.71%)	56 (30.27%)	62 (37.58)
≥Secondary, n (%)	287 (53.54%)	101 (54.30)	105 (56.76)	81 (49.09%)
**Parity, n(%)**				
1–2	215 (39.81%)	78 (41.49%	69 (37.30%)	68 (40.72%)
3	212 (39.26%)	70 (37.23%)	79 (42.70%)	63 (37.72%)
≥4	113 (20.93%)	40 (21.28%)	37 (20.00%)	36 (21.56%)
**Children at 4.5 years**				
Age (month)	55.93±1.38	55.88±1.38	56.03±1.44	55.86±1.32
Hb (g/l)	128.61±13.33	127.68±12.53	129.84±13.45	128.28±14.03
^d^Stunted, n (%)	158 (29.80%)	53 (28.19%)	51 (27.56%)	54 (32.33%)
^d^Underweight, n (%)	209 (39.40%)	75 (39.90%)	73 (39.46%)	61 (36.53%)
^d^Thinness, n (%)	69 (12.78%)	23 (12.23%)	26 (14.05%)	20 (11.97%)
**Children at 9 years**				
Age (month)	106.43±1.50	106.45±1.69	106.42±1.51	106.40±1.26
Hb (g/l)	123.81±9.06	123.37±8.89	124.43±9.49	123.62±8.77
^d^Stunted, n (%)	116 (21.50%)	36 (19.15%)	41 (22.16%)	39 (23.35%)
^d^Underweight, n (%)	215 (39.80%)	77 (40.96%)	75 (40.54%)	63 (37.32%)
^d^Thinness, n (%)	137 (25.50%)	60 (31.91%)	41 (22.16%)	36 (21.56%)

Abbreviation: Hb, hemoglobin; SES, socioeconomic status.: Fe30F, 30mg iron and 400μg folic acid; Fe60F, 60mg iron and 400μg folic acid; MM, Fe30F including 15 micronutrients.

Data are presented as mean ± standard deviation or numbers with percentage in parentheses.

^a^Hb at GW14 (n = 482)

^b^Hb at GW30 (n = 461)

^c^Education: Illiterate, never admitted to school; Primary level, completed 1–5 years of schooling; Secondary level & above secondary level, completed ≥6 years of schooling.

^d^Stunting, underweight, and thinness were defined as children with height for age, weight for age and body mass index (BMI) for age <-2 SD from the median value of height, weight and BMI for age of reference population, respectively.

At 4.5 years of age, about 30% children were stunted, among them, 16% were severely stunted. About 39% children were underweight and among them about 19% were severely underweight. Again, about 13% children were thin, of which 10% were severely thin.

The demographic characteristics of the children in the current study (n = 540) were similar to those of the rest of the group B children (S3 Table) who were available (n = 500) (n = 163 not studied) for the background information from another ongoing study at that time (Tofail F, unpublished).

### Status of nutritional biomarkers

The mean Hb concentration of 9 years old children was 123.81±9.06 g/l (mean±SD) (Table 1). There was no difference in the Hb concentration between boys and girls. The prevalence of anemia was about 15% in 9 years and 5% in 4.5 years old children. Among the 9 years old anemic children, 55% had mild, and 45% had moderate anemia. Severe anemia was not present among the children.

Descriptive statistics of all nutritional biomarkers have been given in Table 2. Folate deficiency (<5.2 nmol/l) was not observed among the study participants; only 5 children (0.93%) had vitamin B_12_ deficiency (<182 pmol/l) (S2 Table). About 13% children had folate (>29.5nmol/l) and 3.1% had vitamin B_12_ concentration (>867pmol/l) above the reference range. Zinc and vitamin A deficiencies were present in 8% and 6.2% children respectively (S2 Table). All studied children were hepcidin deficient when 53.5 μg/l [32] was considered as a cut-off, but only 5 children were deficient when the cut-off was <1μg/l [33]. Iron deficiency was found in 8.0% children when sTfR cut-off was used. However, it was only 0.2% when ferritin cut-off was used (S2 Table). About 2.5% children had IDA according to the defined sTfR cut-off and 0.2% (one child) when applying the ferritin cut-off.

**Table 2:** Plasma biomarkers in school-age children in rural Matlab.

	All children	Fe30F	Fe60F	MM
	(n 540)	(n = 188)	(n = 185)	(n = 167)
sTfR (nmol/l)	44.83±10.96	45.34±11.38	44.67±11.61	44.45±9.69
Ferritin (μg/l)	59.82±31.35	62.04±30.10	59.57±28.55	57.59±35.42
Folate (nmol/l)	23.13±5.74	23.78±5.96	22.53±5.64	23.05±5.57
Vitamin B_12_ (pmol/l)	469.96±174.67	474.88±167.78	460.38±179.18	474.95±177.78
Hepcidin (μg/l)	9.57±5.92	9.85±6.65	9.60±5.69	9.23±5.29
Zinc (μmol/l)	13.33±2.54	13.34±2.55	13.36±2.64	13.29±2.43
Vitamin A (μmol/l)	1.03±0.22	1.03±0.22	1.04±0.23	1.01±0.21

Abbreviation: Fe30F, 30mg iron and 400μg folic acid; Fe60F, 60mg iron and 400μg folic acid; MM, Fe30F including 15 micronutrients; sTfR, soluble transferrin receptor.

Data is presented as mean ± standard deviation.

Based on morphological classification of anemia, about 61% of anemic children (n = 50) and 92% of non-anemic children (n = 431) had normocytic normochromic RBC. About 34% of anemic children and 6.3% of non-anemic children had microcytic RBC while 4.9% of anemic and 1.3% of non-anemic children had normocytic hypochromic RBC.

### Influence of maternal supplementation on child growth and inflammatory markers

Compared to the Fe30F group, children in the Fe60F as well as MM group had significantly higher BAZ scores at 4.5 and 9 years of age (Table 3). However, no significant differences were seen in HAZ or WAZ scores with respect to supplementation. Maternal supplementation did not affect the number of stunted or underweight children either at 4.5 or 9 years of age. However, 9 years old children belonging to the MM and Fe60F groups had fewer thin children (n = 36 and 41, respectively) compared to those in Fe30F group (n = 60, χ^2^ P = 0.02, and 0.04, respectively) (Data not shown).

**Table 3:** Analysis of covariance of child nutritional status and plasma biomarkers in different supplementation groups.

	Fe30F	Fe60F	P-value^1^	MM	P-value^2^
	(n = 188)	(n = 185)		(n = 167)	
**Nutritional status**					
HAZ at 4.5 years	-1.53±0.86	-1.55±0.88	0.59	-1.59±0.87	0.54
HAZ at 9 years	-1.27±0.86	-1.27±0.93	0.98	-1.40±0.88	0.19
WAZ at 4.5 years	-1.76±0.84	-1.78±0.83	0.58	-1.76±0.76	0.98
WAZ at 9 years	-1.68±1.04	-1.67±1.11	0.80	-1.74±0.96	0.38
BAZ at 4.5 years	-1.17±0.82	-1.18±0.76	0.97	-1.11±0.78	0.33
BAZ at 9 years	-1.47±1.09	-1.25±1.08	0.05	-1.23±1.06	0.04
**Biomarkers**					
Hb at 4.5 years	127.76±8.90	129.79±9.49	0.15	128.08±8.77	0.76
Hb at 9 years	123.54±12.50	124.26±13.45	0.52	123.56±14.03	0.98
Folate (nmol/l)	23.66±5.96	22.60±5.64	0.06	20.03±5.57	0.03
ESR (mm/1st hr)	16.15±10.95	15.76±10.43	0.75	14.25^b^±10.28	0.05
CRP (mg/L)	1.36±2.97	0.91±1.60	0.04	0.60±0.63	0.002
RDW-CV (%)	13.67±1.22	13.43±0.96	0.05	13.54±1.05	0.29
MCH (pg)	26.35±2.25	27.50±6.64	0.01	26.64±2.03	0.57

Abbreviation: sTfR, soluble transferrin receptor; Fe30F, 30mg iron and 400μg folic acid; Fe60F, 60mg iron and 400μg folic acid; MM, Fe30F including 15 micronutrients; Hb, hemoglobin, ESR, erythrocyte sedimentation rate, RDW, Red cell distribution width, MCH, mean corpuscular hemoglobin and CRP, plasma concentration of C-reactive protein.

^1^Significant difference between Fe30F and Fe60F

^2^Significant difference between Fe30F and MM

Models were adjusted for socio economic status, body mass index, child sex, mother’s occupation, mother’s education levels and plasma concentration of C-reactive protein

Data are presented as mean ± standard deviation.

When markers of inflammation or infection were considered, children in MM group had significantly lower levels of CRP and ESR compared to the Fe30F group (Table 3). Children in the MM group had significantly lower concentration of plasma folate compared to the Fe30F group.

When considering RBC indices, children in Fe60F group had higher MCH concentration compared to Fe30F group. RDW values were significantly lower in the Fe60F group compared to Fe30F group (Table 3).

### Association of Hb with plasma biomarkers and effect of maternal micronutrient supplementation

In the adjusted linear regression analysis, Hb was significantly inversely associated with plasma sTfR; when the association between Hb and sTfR was stratified by sex the association remained significant only in boys (Table 4). Hb was positively associated with hepcidin in all children and in girls only. Hb was also positively associated with vitamin A in all children (Table 4). A positive tendency was obtained between Hb and Zn in girls, and a positive association was noted between Hb and B_12_ in boys only.

**Table 4:** Linear regression analyses between hemoglobin concentrations and plasma biomarkers in all children and boys and girls separately.

Variables	All supplementation groups					
	All children (n = 540)		Boys (n = 263)		Girls (n = 277)	
	*Adj. β (95% CI)	P	^#^Adj. β(95% CI)	P	^#^Adj. β(95% CI)	P
sTfR (nmol/l)	-0.75(-1.48, 0.01)	0.04	-1.46(-2.38, -0.55)	0.002	0.46(-0.78, 1.70)	0.46
Ferritin (μg/l)	0.13(-0.12, 0.38)	0.31	0.20(-0.15, 0.56)	0.26	0.06(-0.30, 0.43)	0.73
Folate (nmol/l)	-0.11(-1.47, 1.26)	0.87	-1.01(-2.98, 0.96)	0.31	1.03(-0.92, 3.00)	0.30
Vitamin B_12_ (pmol/l)	0.02(-0.02, 0.06)	0.39	0.07(-0.001, 0.14)	0.05	-0.01(-0.07, 0.05)	0.64
Hepcidin (μg/l)	1.274(0.26, 3.31)	0.02	1.12(-1.44, 3.69)	0.38	1.92(0.09, 3.75)	0.04
Zinc (μmol/l)	1.65(-1.44, 4.73)	0.29	-0.85(-5.20, 3.50)	0.70	3.98(-0.48, 8.44)	0.08
Vitamin A (μmol/l)	2.16(0.88, 3.45)	0.001	3.02(1.04, 5.00)	0.003	1.70(0.01, 3.38)	0.04
	Fe60F					
	All children (n = 185)		Boys (n = 87)		Girls (n = 98)	
sTfR (nmol/l)	-0.79(-2.06, 0.48)	0.22	-1.06(-2.74, 0.62)	0.21	-0.5(-3.03, 1.53)	0.51
Ferritin (μg/l)	0.47(-0.02, 0.97)	0.06	0. 72(-0.10, 1.53)	0.08	0.37(-0.27, 1.01)	0.25
Folate (nmol/l)	0.32(-2.22, 2.86)	0.80	-1.06(-4.84, 2.72)	0.57	3.12(-0.72, 6.96)	0.11
Vitamin B_12_ (pmol/l)	0.04(-0.04, 0.12)	0.27	0.19 (0.04, 0.34)	0.01	-0.02(-0.11, 0.07)	0.66
Hepcidin (μg/l)	2.88(0.35, 5.41)	0.02	0.80(-4.31, 5.91)	0.75	4.11(1.20, 7.02)	0.006
Zinc (μmol/l)	-1.05(-6.57, 4.47)	0.70	-5.25(-13.57, 3.07)	0.21	4.29(-4.00, 12.57)	0.30
Vitamin A (μmol/l)	3.13(0.89, 5.38)	0.006	6.04(2.44, 9.64)	0.001	1.11(-1.84, 4.06)	0.45

Abbreviation: Fe60F, 60mg iron and 400μg folic acid; sTfR, soluble transferrin receptor.

Data were given as regression coefficient (β) and 95% confidence intervals

*Adjusted for socio economic status, body mass index, child sex, mother’s occupation, mother’s education levels and plasma concentration of C-reactive protein

^#^Adjusted for socio economic status, body mass index, mother’s occupation, mother’s education levels and plasma concentration of C-reactive protein.

To evaluate the influence of maternal supplementation, the associations between Hb and plasma biomarkers in 9 years old children were stratified by different supplementation groups (Table 4, and S4 Table). In the Fe60F group but not others (Fe30F and MM), Hb was strongly positively associated with plasma hepcidin and vitamin A in all children. When these associations were stratified by sex, Hb was associated with vitamin A in boys and with hepcidin in girls (Table 4). Again in Fe60F group, the significant positive association was noted between Hb and Vitamin B_12_ only in boys (Table 4).

### Long term impact of maternal anemia in school-age children

**Fig 2:**
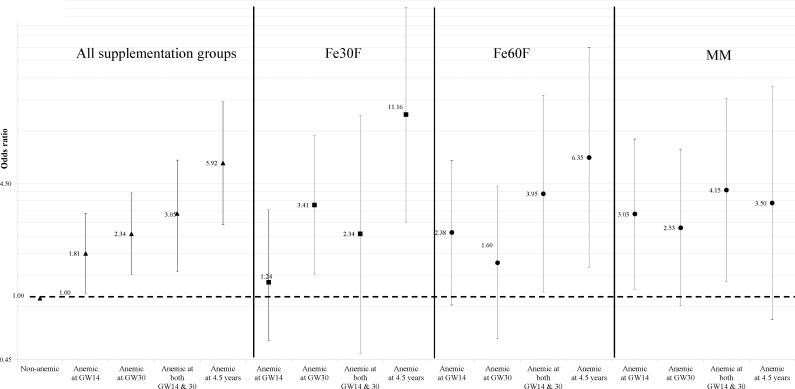
The odds of having anemia at 9 years of age if the mother were anemic at GW-14 or GW-30 or if the children were anemic at 4.5 years of age. Abbreviation: Fe30F, 30mg iron and 400μg folic acid; Fe60F, 60mg iron and 400μg folic acid; MM, Fe30F including 15 micronutrients, GW, Gestational week.

Among mothers during pregnancy, about 28% and 35% of the women at GW14 and GW30, respectively, were found to be anemic, and 23.6% women were anemic at both time points. Percentage of anemia was higher in children at 9 years of age (15%) compared to 4.5 years of age (5%). Notably, thirteen children who were anemic at 4.5 years of age (45%) remained anemic at 9 years of age. Sixty nine children who were non-anemic at 4.5 years became anemic at 9 years. In adjusted model, the odds (OR) of being anemic in 9 years of age was 1.81 (95% CI 1.07, 3.05; P = 0.027) and 2.34 (1.37, 4.00; P = 0.002) fold higher if their mothers were anemic at either GW14 or GW30, respectively, compared to non-anemic mothers (Fig 2). The odds of having anemia at 9 years was even higher if the mothers were anemic at both time points (OR = 3.05, 95% CI 1.42, 6.14; P = 0.002). The odds increased, about 6 times (OR = 5.92, 95% CI 2.64, 13.25; P<0.001) when the children were also anemic at 4.5 years. However, no impact of maternal anemia was observed at 4.5 years of age (at GW14, OR = 0.45, 95% CI 0.15, 1.34; P = 0.15; at GW30, OR = 1.28, 95% CI 0.54, 3.04; P = 0.56 and at both time points, OR = 0.66, 95% CI 0.18, 2.43; P = 0.54).

When the analyses was stratified by supplementation groups, the odds of having anemia at 9 years of age was 2.34 (95% CI 0.49, 11.04; P = 0.28), 3.95 (95% CI 1.09, 14.38; P = 0.03) and 4.15 (95% CI 1.25, 13.76; P = 0.02) times higher in the Fe30F, Fe60F and MM groups, respectively, if their mothers were anemic both at GW14 and GW30 (Fig 2). Again, the odds of having anemia at 9 years of age was 11.16 (95% CI 2.73, 45.59; P = 0.001), 6.35 (95% CI 1.50, 26.81; P = 0.01) and 3.50 (95% CI 0.76, 16.09; P = 0.10) times higher in the Fe30F, Fe60F and MM groups, respectively, if they were anemic at early childhood (4.5 years).

## Discussion

In the present study, we found that maternal supplementation during pregnancy with MM had beneficial effects on child nutritional status (BAZ scores) that also decreased markers of inflammation (CRP, ESR) in 9 years old children.

In a larger cohort of MINIMat children (n = 1634), Khan et al showed that maternal MM supplementation (n = 897) increased the proportion of stunting in boys only at 4.5 years of age [34]. However, we did not find any impact of MM supplementation on the proportion of stunting or underweight children either at 4.5 or 9 years of age, not even in boys. Although, a long-term favorable effect of MM supplementation was seen in terms of fewer numbers of thin children at 9 years, but this was not evident at 4.5 years of age. Data on child growth beyond 5 years of age in relation to maternal supplementation are scarce. A meta-analysis of randomized controlled trials reported that maternal multi-micronutrient supplementation had a significant positive effect on head circumference of under-5 children without any effects on weight, height, WAZ, HAZ and WHZ [35]. In our study, supplementation with MM also markedly reduced markers of inflammation and infection in 9 year old children. The MM supplement of UNICEF/WHO/UNU contained important antioxidants such as vitamin E, vitamin C and selenium which are known to reduce inflammatory responses. It is possible that prenatal MM supplementation improved the immune reserve of the growing fetus that persists and aid in combating infections in later childhood. Very few studies have reported effects of MM supplementation during pregnancy on the health outcomes of older children. Multi-vitamin supplementation of HIV-infected women during pregnancy and lactation was shown to be associated with reduced rate of all types of diarrhea among under-5 HIV-negative children [36]. A study in Nepal demonstrated that maternal vitamin A supplementation enhanced natural antibody concentrations in children at preadolescent age (9 to 13 years); the authors postulated that pre-natal supplementation lead to a higher reservoir and sustained natural immunity in these children [37]. An increasing body of evidence suggests that maternal nutritional status including micronutrients, life-style, exposure to pollutants etc. from preconception through lactation causes fetal or neonatal epigenetic changes that might account for altered mechanisms of growth, metabolism and diseases observed later in life [38].

In the current study, majority of 4.5 (95%) and 9 years (85%) old children, Hb concentration was well within the normal range. The previous report on a larger cohort (n = 1354) of MINIMat children at 4.5 years of age showed similar finding, where 92% of the children had Hb levels within the normal range [39]. The positive association between Hb and plasma concentration of ferritin, B_12_, hepcidin and vitamin A mainly in Fe60F group indicated a beneficial impact of the higher dose of antenatal iron on Hb concentration in 9 years old children. Vitamin A has an important role in iron mobilization into Hb of developing RBC [40,41]. Iron status impacts plasma and liver levels of vitamin A [42]. Supplementation with 60 mg iron and folate during pregnancy increased hepcidin concentration in iron deficient Tanzanian women (serum ferritin ≤12μg/L) [43]. In rural Bangladesh, pregnant women with low iron deficiency, plasma concentrations of Zn, vitamin B_12_, and α-tocopherol were positively associated with Hb levels [44]. We did not find similar associations between Hb and other plasma biomarkers in the Fe30F and MM groups. Hb concentrations did not differ by supplementation groups in children, this finding was in line with the original MINIMat trial where supplementation did not affect Hb concentration in pregnant women at GW30 [14].

The prevalence of anemia in 9 year old children was 15% which was slightly lower than the prevalence rate of 19% obtained in the recent Bangladesh National Micronutrient Survey conducted in school-age (6–11 years) children [4]. Notably, none of the children had folate deficiency; iron (8% or 0.2%) and vitamin B_12_ deficiencies (0.9%) were also minimal suggesting that a major cause of anemia in children was not related to iron, folate or B_12_ deficiency. These results are in line with the findings at 4.5 years of age as reported earlier on MINIMat children [39]. Presence of normocytic normochromic anemia in more than half (about 61%) of the anemic children could indicate presence of recent infections, deficiency of vitamin B_2_ (riboflavin) and genetic disorders such as sickle cell anemia, red blood cell membrane disorders etc. that may cause hemoglobinopathies in children of this age group [45]. Microcytic anemia can be attributed to IDA, vitamin B_6_ (pyridoxine) deficiency and thalassemia [45]. In the current study, the exact nature of anemia (whether genetic or B_2_ or B_6_ deficiency) could not be determined, however, parents were notified, and suspected children were referred to specialists.

Maternal anemia during pregnancy was a strong risk factor for children being anemic at 9 years of age. Several studies have demonstrated that maternal anemia was a risk factor for childhood anemia in under-5 children [41,46]. To the best of our knowledge, this is the first report that shows that this risk is also apparent at 9 years of age. In contrary to other studies, we did not find any association between maternal and childhood anemia at 4.5 years of age. The influence of maternal anemia during pregnancy seems to emerge in pre-adolescent age and not earlier in this cohort. An interesting finding of the present study was that Hb level at 4.5 years of age was a stronger risk factor than maternal Hb (~2 folds higher) for being anemic in 9 years of age. The risk of being anemic at 9 years was higher among the Fe30F group (OR = 11), likely due to low Fe or absence of MM supplementation during pregnancy. The findings reflect that early childhood nutritional status is also very important besides prenatal nutrition for optimum health outcome at later age and highlighting the public health importance of these findings.

One of the limitations of the study was that we could not measure Hb by the cyanmethemoglobin method (the gold standard) although the hemocue technique is well-recognized and suitable for epidemiological surveys. Another drawback was that concentration of multiple micronutrients was not measured in 4.5 year old children or their mothers during pregnancy to compare with the current micronutrient status in school-age children. The strength of our study lies in the fact that we have determined important nutritional markers and indicators of inflammation (CRP) and infection (ESR). The statistical models were adjusted with SES, mother’s education, mother’s occupation, child BMI, child sex, and plasma CRP to rule out possible confounding factors. However, there may be some unmeasured social, environmental, dietary and other factors that may impact the outcomes. Another shortcoming of the current study was that we did not have reliable information on recent morbidity (past 1–2 months). However, we adjusted the statistical models with plasma CRP concentrations as a marker of recent infection.

In conclusion, our results indicate that maternal micronutrient supplementation confers beneficial health effects on pre-adolescent school-age children although the effects were not apparent at an earlier age of 4.5 years. Anemia during pregnancy and in early childhood appeared to be important risk factors for anemia in pre-adolescent age. This information might aid the public health services to take initiatives aimed at reducing anemia and micronutrient deficiencies in early childhood in addition to the programs being carried out for women during pregnancy. Further studies are needed to better understand the mechanisms by which maternal and early childhood nutritional status influence health in later childhood.

## Supporting Information

S1 Table External quality assurance program (VITAL EQA) of Centers for Disease Control and Prevention. (DOCX)

S2 Table Indicators used in this study to define anemia and micronutrient deficiencies in school-age children. (DOCX)

S3 Table Descriptive statistics of group B children who were included and those not included in the current study. (DOCX)

S4 Table Linear regression analyses between hemoglobin concentrations and plasma biomarkers in children in Fe30F and MMS supplementation groups. (DOCX)
