# Supplementary material for: Maternal Micronutrient Supplementation and Long Term Health Impact in Children in Rural Bangladesh
Source: PLoS One. 2016 Aug 18;11(8):e0161294. doi: 10.1371/journal.pone.0161294 (PMC4990280; doi:10.1371/journal.pone.0161294)
Supplement: S1 Table — (DOCX) [file pone.0161294.s001.docx]

**S1 Table: External quality assurance program (VITAL EQA) of Centers for Disease Control and Prevention.**

|  |  | Retinol | Ferritin | sTfR | CRP | Folate | Vitamin B12 |
| --- | --- | --- | --- | --- | --- | --- | --- |
| Level 1 | CDC target value | 39.0±2.1 | 34.3±1.4 | 4.8±0.1 | 1.1±0.1 | 3.9±0.4 | 411.0±14.5 |
|  | Our observed value | 39.1±1.9 | 40.3±0.1 | 4.9±0.1 | 1.3±0.01 | 3.9±0.1 | 409.0±6.9 |
| Level 2 | CDC target value | 43.5±3.3 | 36.5±1.7 | 4.9±0.1 | 1.2±0.1 | 4.7±0.4 | 521.0±13.0 |
|  | Our observed value | 43.3±1.4 | 38.1±0.9 | 4.9±0.2 | 1.5±1.2 | 3.9±0.1 | 509.0±10.7 |
| Level 3 | CDC target value | 51.7±1.8 | 86.7±3.4 | 5.6±0.2 | 5.67±0.1 | 6.3±0.6 | 768.0±30.5 |
|  | Our observed value | 53.2±1.7 | 96.5±1.3 | 5.7±0.1 | 7.1±0.0 | 4.7±0.1 | 743.0±15.4 |
