# Supplementary material for: Maternal Micronutrient Supplementation and Long Term Health Impact in Children in Rural Bangladesh
Source: PLoS One. 2016 Aug 18;11(8):e0161294. doi: 10.1371/journal.pone.0161294 (PMC4990280; doi:10.1371/journal.pone.0161294)
Supplement: S2 Table — (DOCX) [file pone.0161294.s002.docx]

**S2 Table**. **Indicators used in this study to define anemia and micronutrient deficiencies in school-age children.**

| Condition | Indicator | Cut-off |
| --- | --- | --- |
| Anemia | Hb | < 115 g/l [[1](#_ENREF_1)] |
| Iron deficiency (ID) | Plasma ferritin or  Plasma sTfR | <15 µg/l [[1](#_ENREF_1)] or  >59 nmol/l [[2](#_ENREF_2)] |
| Iron deficiency anemia (IDA) | Plasma ferritin and Hb or  Plasma sTfR and Hb | <15 µg/l and <115 g/l [[1](#_ENREF_1)] or  >59 nmol/l and <115 g/l [[1](#_ENREF_1),[2](#_ENREF_2)] |
| Folate deficiency | Plasma folate | <5.2 nmol/l [[3](#_ENREF_3)] |
| Vitamin B_12_ deficiency | Plasma vitamin B_12_ | < 182 pmol/l [[3](#_ENREF_3)] |
| ^*^Hepcidin deficiency | Plasma hepcidin | <1 µg/l [[4](#_ENREF_4)]  <53.5 µg/l [[5](#_ENREF_5)] |
| Zinc deficiency | Plasma zinc | <9.9 µmol/l [[6](#_ENREF_6)] |
| Vitamin A deficiency | Plasma vitamin A | <0.7 µmol/l [[7](#_ENREF_7)] |
| Normocytic normochromic  anemia | MCV and MCH | 74.4-93.9 fl and 24.3-31.8 pg [[8](#_ENREF_8)] |
| Microcytic anemia | MCV | <74.4 fl |

1. WHO (2001) Iron deficiency anaemia: assessment, prevention, and control. A guide for programme managers. Geneva.

2. Kolbe-Busch S, Lotz J, Hafner G, Blanckaert NJ, Claeys G, et al. (2002) Multicenter evaluation of a fully mechanized soluble transferrin receptor assay on the Hitachi and cobas integra analyzers. the determination of reference ranges. Clin Chem Lab Med 40: 529-536.

3. Akcam M, Ozdem S, Yilmaz A, Gultekin M, Artan R (2007) Serum ferritin, vitamin B(12), folate, and zinc levels in children infected with Helicobacter pylori. Dig Dis Sci 52: 405-410.

4. EIA-5258 E DRG Hepcidin-25 (bioactive)

5. Sdogou T, Tsentidis C, Gourgiotis D, Marmarinos A, Gkourogianni A, et al. (2015) Immunoassay-based serum hepcidin reference range measurements in healthy children: differences among age groups. J Clin Lab Anal 29: 10-14.

6. IZiNCG (2007) International Zinc Nutrition Consultative Group. Assessing population zinc status with serum zinc concentration.

7. WHO (2011) Serum retinol concentrations for determining the prevalence of vitamin A deficiency in populations. Vitamin and Mineral Nutrition Information System. Geneva.

8. NHANES.CDC (2003–2004) Laboratory Procedure Manual.Complete Blood Count (CBC) with Five-Part Differential.
