# Supplementary material for: Maternal Micronutrient Supplementation and Long Term Health Impact in Children in Rural Bangladesh
Source: PLoS One. 2016 Aug 18;11(8):e0161294. doi: 10.1371/journal.pone.0161294 (PMC4990280; doi:10.1371/journal.pone.0161294)
Supplement: S3 Table — (DOCX) [file pone.0161294.s003.docx]

**S3 Table:** Descriptive statistics of the studied children and the children who were not studied.

|  | Group B children* | | |
| --- | --- | --- | --- |
| Variables | | Included in current study (n=540) | Not included in the study (n=500) |
| Child Sex | |  |  |
| Boys | | 263 (48.7%) | 248 (49.6%) |
| Girls | | 277 (51.3%) | 252 (50.4%) |
| HAZ | | -1.31±0.89 | -1.44±0.86 |
| WAZ | | -1.70±1.04 | -1.71±0.96 |
| BAZ | | -1.32±±1.08 | -1.22±1.09 |
| BMI | | 14.27±1.63 | 14.57±1.68 |
| SES | |  |  |
| 1^st^ tertile, n (%) | | 180 (33.46%) | 153 (30.6%) |
| 2^nd^ tertile, n (%) | | 179 (33.27%) | 162 (32.4%) |
| 3^rd^ tertile, n (%) | | 179 (33.27%) | 185 (37.0%) |

Abbreviation: HAZ, height-for-age; WAZ, weight-for-age; BMI, body mass index, BAZ, BMI-for-age; and SES, Socio economic status.

*A total of n=2735 MINIMat children were studied at the age of 4.5 years. Group A children (n=1432) were born between April 2002 to June 2003. Group B (n=1303) were born between June 2003 to June 2004.
