# Supplementary material for: Maternal Micronutrient Supplementation and Long Term Health Impact in Children in Rural Bangladesh
Source: PLoS One. 2016 Aug 18;11(8):e0161294. doi: 10.1371/journal.pone.0161294 (PMC4990280; doi:10.1371/journal.pone.0161294)
Supplement: S4 Table — (DOCX) [file pone.0161294.s004.docx]

**S4 Table**. **Linear regression analyses between hemoglobin concentrations and plasma biomarkers in children in Fe30F and MM supplementation groups.**

|  | Fe30F | | | | | |
| --- | --- | --- | --- | --- | --- | --- |
|  | All children (n=188) | | Boys (n=95) | | Girls (n=93) | |
| Variables | ^*^Adj. β (95% CI) | p | ^#^Adj. β(95% CI) | p | ^#^Adj. β(95% CI) | p |
| sTfR (nmol/l) | -0.82(-2.02, -0.37) | 0.17 | -2.54(-3.95, -1.14) | 0.001 | 2.24(0.23, 4.26) | 0.02 |
| Ferritin (µg/l) | -0.27(-0.71, 0.17) | 0.22 | -0.41(-1.14, 0.32) | 0.26 | -0.15(-0.73, 0.42) | 0.59 |
| Folate (nmol/l) | -0.93(-3.15, 1.30) | 0.41 | -0.75(-4.02, 2.52) | 0.64 | 0.39(-2.87, 3.66) | 0.81 |
| Vitamin B_12_ (pmol/l) | -0.03(-0.11, 0.05) | 0.40 | -0.01(-0.14, 0.12) | 0.83 | -0.04(-0.15, 0.07) | 0.45 |
| Hepcidin (µg/l) | -0.26(-2.40, 2.91) | 0.85 | 0.41(-4.29, 5.12) | 0.17 | -0.66(-2.68, 4.00) | 0.69 |
| Zinc (µmol/l) | 2.45(-2.80, 7.69) | 0.35 | -0.54(-7.27, 6.19) | 0.87 | 5.64(-3.04, 14.31) | 0.22 |
| Vitamin A (µmol/l) | 1.41(-0.79, 3.62) | 0.20 | 2.96(-0.36, 6.27) | 0.08 | 0.43(-2.63, 3.50) | 0.77 |
|  | MM^a^ | | | | | |
|  | All children (n=67) | | Boys (n=81) | | Girls (n=86) | |
| sTfR (nmol/l) | -0.53(-2.00, 0.93) | 0.47 | -1.08(-2.96, 0.79) | 0.25 | -0.37(-2.02, 2.75) | 0.76 |
| Ferritin (µg/l) | 0.17(-0.23, 0.58) | 0.39 | 0.26(-0.21, 0.73) | 0.27 | -0.18(-1.01, 0.65) | 0.66 |
| Folate (nmol/l) | 0.02(-2.52, 2.56) | 0.98 | -0.50(-4.05, 3.04) | 0.77 | 0.32(-3.50, 4.14) | 0.86 |
| Vitamin B_12_ (pmol/l) | 0.05(-0.03, 0.13) | 0.25 | 0.06(-0.05, 0.16) | 0.29 | 0.05(-0.09, 0.18) | 0.47 |
| Hepcidin (µg/l) | 1.89(-1.08, 4.86) | 0.21 | 5.06(0.70, 9.43) | 0.02 | -0.34(-4.43, 3.74) | 0.86 |
| Zinc (µmol/l) | 4.78(-0.86, 10.42) | 0.09 | 4.18(-4.34, 12.69) | 0.33 | 3.80(-4.04, 11.64) | 0.33 |
| Vitamin A (µmol/l) | 1.37(-0.96, 3.70) | 0.24 | -0.13(-3.54, 3.28) | 0.94 | 2.83(-0.45, 6.11) | 0.09 |

Abbreviation: sTfR, soluble transferrin receptor; Fe30F, 30mg iron and 400μg folic acid

Data were given as regression coefficient (β) and 95% confidence intervals;

*Adjusted for socio economic status, body mass index, child sex, mother’s occupation, mother’s education levels and plasma concentration of C-reactive protein;

^#^ Adjusted for socio economic status, body mass index, mother’s occupation, mother’s education levels and plasma concentration of C-reactive protein

^a^MM; 30 mg of iron, 400 μg of folic acid, 800 μg of vitamin A, 200 IU of vitamin D, 10 mg of vitamin E, 70 mg of vitamin C, 1.4 mg of vitamin B_1_, 1.4 mg of vitamin B_2_, 18 mg of niacin, 1.9 mg of vitamin B_6_, 2.6 μg of vitamin B_12_, 15 mg of zinc, 2 mg of copper, 65 μg of selenium, and 150 μg of iodine. [[1](#_ENREF_1)]

1. Persson LA, Arifeen S, Ekstrom EC, Rasmussen KM, Frongillo EA, et al. (2012) Effects of prenatal micronutrient and early food supplementation on maternal hemoglobin, birth weight, and infant mortality among children in Bangladesh: the MINIMat randomized trial. *JAMA* 307: 2050-2059.
